# Contrast-Enhanced Ultrasonography for Differential Diagnosis of Benign and Malignant Thyroid Lesions: Single-Institutional Prospective Study of Qualitative and Quantitative CEUS Characteristics

**DOI:** 10.1155/2022/8229445

**Published:** 2022-04-23

**Authors:** Hana Petrasova, Radka Slaisova, Tomas Rohan, Karel Stary, Jitka Kyclova, Tomas Pavlik, Petra Kovalcikova, Tomas Kazda, Vlastimil Valek

**Affiliations:** ^1^Department of Radiology and Nuclear Medicine, University Hospital Brno, Brno 625 00, Czech Republic; ^2^Faculty of Medicine, Masaryk University, Brno 656 53, Czech Republic; ^3^Department of Gastroenterology, University Hospital Brno, Brno 625 00, Czech Republic; ^4^The Institute of Pathology, University Hospital Brno, Brno 625 00, Czech Republic; ^5^The Institute of Biostatistics and Analyses, Faculty of Medicine, Masaryk University Brno, Brno 602 00, Czech Republic; ^6^Department of Radiation Oncology, Masaryk Memorial Cancer Institute and Faculty of Medicine, Masaryk University, Brno 656 53, Czech Republic

## Abstract

**Objectives:**

To extend and revise the diagnostic value of contrast-enhanced ultrasonography (CEUS) for differentiation between malignant and benign thyroid nodules.

**Methods:**

This single-institution prospective study aims to compare CEUS qualitative and objective quantitative parameters in benign and malignant thyroid nodules. Consecutive cohort of 100 patients was examined by CEUS, 68 out of them were further analysed in detail. All included patients underwent cytological and/or histopathological verification of the diagnosis.

**Results:**

Fifty-five (81%) thyroid nodules were benign, and 13 (19%) were malignant. Ring enhancement pattern was strongly associated with a benign aetiology (positive predictive value 100%) and heterogeneous enhancement pattern with malignant aetiology (positive predictive value 72.7%). The shape of the TIC (time-intensity curve) was more often identical in the benign lesion (98.2%) than in malignant lesions (69.2%), *p*=0.004.

**Conclusions:**

This study indicates that CEUS enhancement patterns were significantly different in benign and malignant lesions. Ring enhancement was a very strong indicator of benign lesions, whereas heterogeneous enhancement was valuable to detect malignant lesions.

## 1. Introduction

Routine use and technical advances in ultrasound imaging have led to an increase in the number of thyroid nodules detected. The use of high-resolution ultrasonography may be associated with incidental detection of thyroid nodules in up to 67% of the general population [1]. The vast majority of all thyroid nodules are benign, 10%–15% are follicular adenomas and only 5–9% are malignant what makes a differential diagnosis of suspected nodules of high clinical importance [2–7]. Gray-scale ultrasound characteristics are commonly used for the assessment of thyroid nodules. Size, localization, composition, echogenicity, shape, margins, presence of echogenic foci, color, and spectral Doppler are all evaluated in establishing a differential diagnosis. Conventional sonographic imaging has high sensitivity but relatively low specificity (58.5%) [7–10]. No individual conventional US characteristic can unequivocally distinguish benign and malignant thyroid nodules. Numerous classification systems for evaluating thyroid nodules on ultrasound are used to determine the need for fine-needle aspiration (FNA), which is usually considered to be the gold standard for confirming the nature of thyroid nodules [9, 11]. Nevertheless, this determines an elevated number of FNA also in benign lesions. Thus, ongoing research on new ultrasound techniques such as contrast-enhanced ultrasonography (CEUS) and elastography aims to increase the specificity and the diagnostic accuracy for detection of thyroid malignancies.

Nowadays, CEUS is established as an effective technique to evaluate microvascularization. Depiction of tumor microvascularization by CEUS may be more accurate than changes assessable by Doppler ultrasound. CEUS is increasingly used in nearly all organs [12–17]. The EFSUMB (European Federation of Societies for Ultrasound in Medicine and Biology) guidelines for the use of CEUS on nonliver application published in 2017 consider CEUS as a promising noninvasive method for the differential diagnosis of the benign and malignant thyroid nodules, but further studies are needed to explore reliable diagnostic standards [12, 13].

This single-institution prospective study aims to further extend previous reports in this research field, with the study of diagnostic value of CEUS based on qualitative (characterize nodule vascularization compared to the normal surrounding tissue) and objective quantitative enhancement analysis. Quantitative evaluation is based on parameters of time-intensity curves (TIC) that are generated by placing the region of interest (ROI) into the nodule and comparing it to the surrounding tissues utilizing dedicated software. The other aim was to provide in discussion review of the impact of relevant CEUS parameters.

## 2. Methods

### 2.1. Patients' Selection

A consecutive cohort of 100 patients indicated to conventional thyroid US examination in the period from May 2016 to July 2018 was screened for eligibility for this prospective study. Inclusion criteria consist of the presence of focal thyroid lesion greater than 8 mm measured in B mode using the linear probe and willingness to undergo CEUS examination immediately after standard conventional US. Only single nodules were included in order to prove that sampling by FNA (fine-needle aspiration) or histopathology concerns this nodule of our choice. Possible basic disease of the thyroid gland did not affect the inclusion or noninclusion of the lesion into the study.

Exclusion criteria included age less than 18 years, pregnancy, general contraindication of CEUS, noncooperating patient, cystic lesion with no solid component (purely cystic lesions with anechoic content, sharply demarcated with/without echogenic foci with comet tail artifact), or confusing terrain of multiple merging nodules occurring throughout the gland (Table 1).

Study design and protocol were approved by the Institutional Ethics Committee (05–110516/EK). All patients have given their written informed consent before the CEUS examination. The patient was instructed not to move and breathe normally, regularly.

### 2.2. Ultrasound Examination

All sonographic examinations were performed using US scanner Philips iU22 (Philips Healthcare, the Netherlands) equipped with linear probe L12-5. Standard machine presets (small parts thyroid for conventional US examination and contrast general for contrast-enhanced ultrasonography) were used. All patients were examined in the supine position by one experienced radiologist (HP, 10 years of experience) performing thyroid ultrasound routinely.

The plane with the maximal lesion size and corresponding amount of surrounding thyroid tissue was chosen for CEUS, and the transducer was switched into side-by-side power modulation CEUS mode with a low mechanical index (MI 0.07). The focus was always placed deeper than the examined nodule to prevent microbubble disruption. Once set, US parameters preserved unchanged throughout the examination for each patient.

Via peripheral venous cannula in the cubital region, the second generation of microbubble contrast, sulfur hexafluoride encapsulated by phospholipid membrane (SonoVue, Bracco International, Milan, Italy), was applied in a dose of 2.4 mL as a bolus, immediately followed by 10 mL saline flush. Meanwhile, the timer on machine was turned on, and the selected plane was kept stable. A real-time video loop was digitally recorded for 2 minutes in each patient. Recorded data were blinded and digitally stored in raw format on an external hard disk connected to a computer workstation with quantification software.

After the inclusion of the last patient, CEUS video loops were reviewed in a random order by an experienced radiologist, who was blinded to the patients' clinical data, history, and FNA findings.

### 2.3. Ultrasound Qualitative Analysis

Characteristics used for qualitative analysis include characterization of the enhancement pattern (homogeneous, heterogeneous, ring enhancement, and absent enhancement), characterization of time-intensity curves shape (monophasic, typical biphasic, atypical biphasic, and polyphasic), and comparison of the curve shape of the lesion and the surrounding tissue (identical and different). These characteristics are in detail described in Appendix A (Table 2).

### 2.4. Ultrasound Quantitative Analysis

Time-intensity curves (TIC) for quantitative analysis were obtained by postprocessing of the digital data in dedicated quantification software (QLab©, version 13, Philips, the Netherlands). ROI for thyroid nodule covered the entire lesion if feasible or maximum possible area. For surrounding thyroid parenchyma, the shape and size of nodule ROI were copied and placed into thyroid tissue outside the lesion area. If the ROI of the nodule was too large, the maximum possible area of the surrounding parenchyma was covered. The software automatically calculated and displayed TICs. The curve-fitting equation to the waveform was applied. QLab software provides several curve fits. The model called local density random walk wash-in/wash-out (LDRW WIWO) was selected based on the R2 or Chi2 values for the level of accuracy. In addition, the following software functions were used to obtain the most objective values: Active motion compensation© and set background© which are in detail described in Appendix B. In the presented study, LDRW WIWO curve fitting was selected for all patients and derived parameters were automatically calculated by software—PI (peak intensity), TFPTOH (time from peak to one half), WIS (wash in slope), and TTP (time to peak). Additional corresponding indexes (the parameter value in nodule divided by the value in surrounding parenchyma) were calculated (PI index, TFPTOH index, WIS index, and TTP index).

Two more software-provided parameters (echo standard deviation and echo mean) were used to calculate the heterogeneity value (HV) according to the formula standard deviation/mean intensity ×100 [18, 19]. HV of the thyroid nodule and surrounding parenchyma was calculated at the time of peak—HVp [19]. The heterogeneity ratio (HR) is calculated as the ratio of the HV of the nodule to that of the surrounding parenchyma [18, 19]. HR was calculated for peak time—HRp. All evaluated parameters are in detail described in Appendix C (Table 3).

### 2.5. Histological Verification

All included patients underwent the US-guided FNA and/or surgical thyroidectomy to prove the diagnosis. If both methods were performed, the final reference standard was considered to be the histopathological finding after surgery. All specimens were histologically classified by an experienced pathologist who was blinded to US findings. Nodules with benign cytology diagnosis at FNA that did not undergo surgery were regarded benign when showed stable findings on the follow-up US examinations for at least 2 years. This situation occurred in a total of 20 patients.

### 2.6. Statistical Analysis

Basic descriptive statistics (mean, median, minimum, and maximum) were used to summarize the observed continuous (quantitative) parameters. Differences in these parameters according to the type of tumor were evaluated using the Mann–Whitney test. The categorical (qualitative) parameters were summarized using absolute and relative frequencies and compared by Fisher's exact test. The analysis of the receiver operating characteristic (ROC) curves was used to assess the quantitative CEUS parameters to differentiate malignant nodules from benign nodules. The area under a curve (AUC) and the optimal cut-off values corresponding to the maximum Youden index were calculated. Sensitivity, specificity, positive predictive value (PPV), negative predictive value (NPV), and overall accuracy with 95% confidence intervals for these optimal cut-off values were determined.

All data analysis was executed using the Statistical Package for the Social Sciences software (version 25.0, IBM-SPSS, Armonk, NY, USA) and *R* Statistical software (version 3.4.4, *R* Foundation for Statistical Computing, Vienna, Austria). A *p* value of 0.05 or less was considered statistically significant.

## 3. Results

### 3.1. Patients' Characteristics

**Figure 1:**
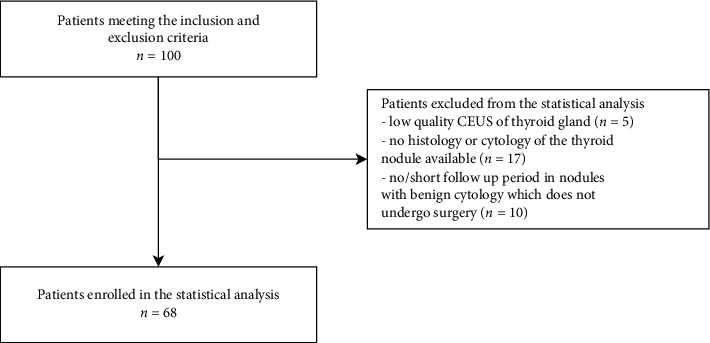
Flowchart demonstrating patients included in the statistical analysis.

Out of total 100 included patients, 32 patients were not further analysed because of the following reasons: image quality of CEUS was unsatisfactory; no histology or cytology of the thyroid nodule was available; and no/short follow-up period for nodules with benign cytology diagnosis that did not undergo surgery because of the patient's reluctance to continue the research. Thus, a total of 68 patients were included in statistical analysis (54 women, 79%; median age 54 years) with 68 thyroid nodules (median size 24 mm, range 9–99 mm), Figure 1. Patients' characteristics are presented in Table 4.

Among 68 thyroid nodules included in this study, 48 nodules (48/68; 71%) were surgically removed. Thirty-seven (37/48; 77%) were benign (29 nodular goiters, 6 follicular adenomas, 1 oncocytic adenoma, and 1 fibrous variant of Hashimoto disease), and 11 (11/48; 23%) were malignant (9 papillary thyroid carcinomas, 1 follicular carcinoma, and one metastasis of colorectal carcinoma). Cytologic diagnosis revealed 18 cases of benign lesions (category II of The Bethesda System for Reporting Thyroid Cytopathology) and 2 cases of malignant lesions (1 papillary thyroid carcinoma (the patient did not undergo surgery due to other comorbidities), 1 diffuse large B-cell lymphoma (DLBCL; confirmed by autopsy)). In the subgroup of solid thyroid nodules, a total of 33 (33/44; 75.0%) were benign.

No significant difference in size between benign and malignant nodules was found (benign lesions size range 9 to 99 mm and malignant lesions size range 10 to 67 mm, *p*=0.773). Patients with malignant lesions did not differ significantly from patients with benign lesions in either gender (*p*=0.122), age (*p*=0.106), or lesion localization (*p*=0.189).

### 3.2. Qualitative Ultrasound Analysis

**Figure 2:**
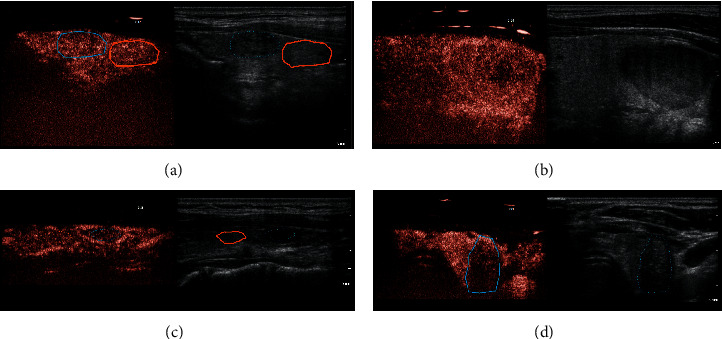
Enhancement patterns on CEUS. (a) The homogeneous enhancement pattern of the lesion, (b) ring enhancement with the heterogeneous central portion, (c) ring enhancement with the homogeneous central portion, and (d) heterogeneous enhancement.

The enhancement patterns of benign and malignant nodules were statistically different (*p* < 0.001). Patients with a malignant lesion were more likely to have heterogeneous enhancement than patients with a benign lesion (69.2% vs. 7.3%), and no patient with a malignant lesion had ring enhancement. Enhancement patterns are presented in and showed in Figure 2. Twenty-seven of the 55 benign nodules (49.1%) showed ring enhancement, and 9 of 13 malignant nodules (69.2%) showed heterogeneous enhancement. Ring enhancement has a sensitivity of 49%, specificity of 100%, positive predictive value (PPV) of 100%, negative predictive value (NPV) of 32%, and the accuracy of 59% for detection of a benign lesion. The heterogeneous pattern has a sensitivity of 69%, specificity of 93%, PPV of 69%, NPV of 93%, and the accuracy of 88% for detection of malignant lesions. The enhancement pattern of lesions with solid composition was homogeneous, heterogeneous, and ring in 18, 11, and 15 cases, respectively. Corresponding values for lesions with mixed composition were 8, 1, and 8 cases, for lesions with spongiform composition 2, 1, and 4, respectively.

**Figure 3:**
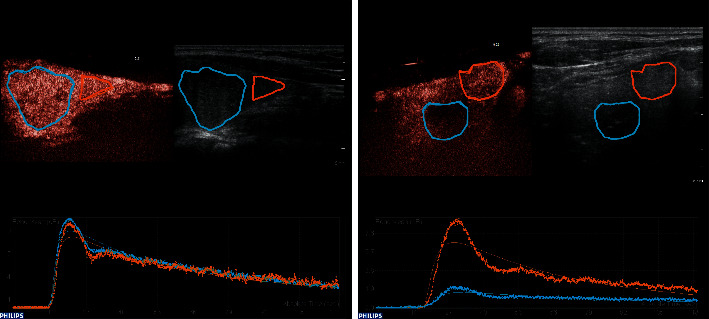
Time-intensity curve shapes. (a) The identical TICs shapes within the benign lesion (blue ROI) and surrounding parenchyma (orange ROI) and (b) the different TICs shapes within the malignant lesion (blue ROI; papillary thyroid carcinoma) and surrounding parenchyma (orange ROI).

The shape of the TIC curve in the thyroid lesion does not differ significantly corresponding to the biological nature of the lesion (*p*=0.275). The shape of the TIC curves of the nodule and the surrounding tissue was significantly different (*p*=0.004) with the shape of the TIC curves being more often identical in benign (98.2%) than in malignant lesions (69.2%), Figure 3Table 5.

### 3.3. Quantitative Ultrasound Analysis

The study did not show a statistically significant difference in any of the monitored quantitative parameters concerning the biological nature of the lesion (AUC 50–65% and *p* = 0.432–0.941). ROC analysis of quantitative characteristics is summarized in Appendix D (Table 6).

## 4. Discussion

An increasing thyroid malignancy incidence (around 8 per 100 000 in Europe) [20] brings the need to extend the noninvasive diagnostic method that allows reliable differentiation between malignant and benign thyroid nodules, superior to the current B-mode US features [21].

Our study was focused to extend available scientific data regarding CEUS for differentiation between malignant and benign thyroid nodules. The aim was to find the CEUS characteristics of malignant and also benign lesions, to compare each other, and to find differences.

This aim of our study was supported by the EFSUMB guidelines [13], which state the need for further studies to explore reliable diagnostic standards in this field.

The most relevant finding in our study was confirmation that ring enhancement can determine benign thyroid nodules with the specificity of 100% and a positive predictive value of 100% in our cohort which is supported by other reports [13]. Ring enhancement is probably associated with capsular and peripheral compressed parenchymal vessels around the lesion. In addition, Zhang et al. found that ring enhancement was associated with a benign aetiology of thyroid nodule (sensitivity: 83.0%, specificity: 94.1%, and accuracy: 88.5%), which the authors explain by compressing parenchymal vessels around the lesion [22]. Attention must be paid to careful evaluation of the ring as Cantisani et al. described incomplete or interrupted ring enhancement as a sign of malignancy [23].

Another statistically significant finding in our study was a heterogeneous enhancement pattern for the detection of malignant lesions (*p* < 0.001) with the sensitivity of 69.2% and accuracy of 88.2%. This finding is in concordance with the data published in the EFSUMB guidelines and by Zhang et al., where the heterogeneous contrast enhancement pattern in the diagnosis of thyroid malignancy has a sensitivity, specificity, and accuracy of 88.2%, 92.5%, and 90.4%, respectively [22]. In addition, Yuan et al. and Wu et al. demonstrated heterogeneous enhancement as a statistically significant indicator of thyroid malignancy (*p* < 0.001) [24, 25].

In purely cystic lesions with no solid component (TI-RADS 1 category), malignancy can be convincingly excluded by the imaging. Thus, these kinds of lesions were not enrolled in our study. In our study, not a single solid lesion showed absent enhancement. On the contrary, Wu et al. observed a nonenhancement pattern in 41.9% of solid thyroid nodules [25]. This pattern was confirmed to be indicative of benignancy; compared to us, they used a half dose of SonoVue contrast agent (2.4 mL vs. 1.2 mL) [25]. In addition, Bartolotta et al. described a nonenhancement pattern in 30.7% of malignant thyroid nodules [26]. The authors themselves hypothesized that the data could be very well related to the size of the lesion rather than to the histology [26]. In their study, nodules measuring less than 1 cm showed mainly absent vascularization, nodules with a diameter larger than 2 cm presented diffuse contrast enhancement, irrespective of the histology [26].

Most mixed nodules (based on the internal ultrasound composition) are benign with a reported incidence of malignancy of about 14% [10]. In our study, 2 of 17 mixed nodules (12%) were malignant (DLBCL and follicular carcinoma).

In the solid nodules subcategory, there is generally a higher probability of malignancy. In our study, 11 of 44 nodules (25%) were malignant. Our study confirmed that in the solid nodules subgroup, ring enhancement was strongly linked to a benign aetiology (PPV 100%) and heterogeneous enhancement was linked to malignancy (PPV 72.7%). Patients with a solid malignant lesion were more likely to have heterogeneous enhancement than patients with a benign solid lesion (72.7% vs. 9.1%). With that, our study suggests that the ring enhancement pattern was very worthwhile in identification of benign lesions, whereas the heterogeneous enhancement pattern was valuable in detection of malignant lesions. According to these findings, heterogeneous enhancement of thyroid nodules could be an indicator for FNA to verify the diagnosis.

The EFSUMB guidelines include TIC shapes among the discriminating characteristics. Time-intensity curve patterns of wash-out may appear as polyphasic or monophasic related to the heterogeneity of the nodule and histology, with a sensitivity, specificity, and accuracy of 76.9%, 84.8%, and 82.6%, respectively [13]. In the present study, the shape of the TIC curve in the thyroid lesion does not differ significantly (*p*=0.275) corresponding to the biological nature of the lesion, although some authors observed monophasic wash-out curves in 93% of benign lesions and polyphasic wash-out curves in 89% of malignant lesions [27]. To the best of our knowledge, unique is the comparison of the TIC shape in the nodule and in the surrounding tissue. Surprisingly, this is even though the shape of the curve is easily available for any TIC analysis. In our study, a significant difference was reached by comparison of the TIC shapes of the nodule and the surrounding tissue. The shape of the TIC curves was more often identical in the benign lesion (98.2%) than in malignant lesions (69.2%), *p*=0.004. The evaluation of this parameter will need to be verified on a larger group of patients.

Hypoenhancement is used to be mentioned as a strong predictor of malignancy on CEUS with high sensitivity, specificity, and accuracy of 82%, 85%, and 84%, respectively [28 22]. In the study of Deng et al., 82.1% (46 of 56) of papillary thyroid carcinoma showed hypoenhancement [28]. This is attributed to the lack of blood supply in papillary thyroid carcinoma [28]. Similarly, Yuan et al. proved lower enhancement compared to the surrounding gland as an aspect with extremely high specificity (95.12%) [24]. Controversially, other studies reported that malignant nodules present a higher internal vasculature (52.3%) than benign nodules (14.3%) [29] and higher vascular density was found in malignant nodules [30]. In our study, we proceeded with objective quantitative measurement of signal intensity using the peak-intensity parameter. We did not observe any statistically significant difference in the absolute values of peak intensity (benign nodules 1.9–15.0 dB and malignant nodules 1.5–12.9 dB, *p*=0.467) also in the time to peak (benign nodules 9.5–38.7 sec and malignant nodules 14.0–32.4 sec, *p*=0.780). AUC reached 50.8% and 57.1%, respectively. Similarly, insignificant difference was achieved by the parameters PI index and TTP index. Nemec et al. also did not found a significant difference in absolute peak enhancement and time to peak [31]. Wu et al. study brought significant results by the monitoring of the relative arrival time and the relative wash-out time [25].

According to the area under the ROC curve, the quantitative parameters of CEUS and parameters related to echo mean and echo standard deviation (heterogeneity value and heterogeneity ratio) were insufficiently discriminating the aetiology of focal thyroid lesions (AUC ranges between 50 and 65%, *p* = 0.432–0.941). In addition, a meta-analysis of seven eligible studies showed that quantitative evaluation was inferior to qualitative analysis for the differentiation of the benign and the malignant thyroid nodules [32].

Although prospective, our study has several limitations. Generally, there is a disagreement about the clinical value of CEUS on the thyroid in the present literature [25]. Overlapping data in the published literature regarding qualitative and quantitative evaluation and indeterminate criteria of benign and malignant nature of thyroid nodules indicate a limitation in the interpretation [13]. So far, there is no established standard for examination methodology; thus, it is difficult to compare interinstitutional results.

No single indicator seems to be sensitive or specific sufficiently. However, the combination of several indicators could increase the diagnostic accuracy. Therefore, the results should be interpreted with respect to the clinical data, the conventional US, and other findings to improve diagnostic accuracy in the assessment of thyroid nodules [13, 24, 25, 28]. The interobserver variability was not analysed in the study.

Another limitation is the need to determine some size of evaluated lesions as a part of inclusion/exclusion criteria. Generally, nodules >1 cm are evaluated, since they have a greater potential to be clinically significant cancers, but occasionally, even nodules that are 7–10 mm occasionally require further evaluation because of suspicious features [21]. According to our unpublished pilot data, we experienced technical difficulties in performing CEUS for thyroid nodules in lesions smaller than 8 mm such as difficulties in ROI placement, low quality of recorded video loops, and untrustworthy results of quantitative CEUS analysis. Therefore, lesions of the greatest diameter ≤8 mm were excluded from the study. The CEUS technique will probably still be insufficient for differentiating small foci of microcarcinoma.

Finally, in a routine setting, CEUS is unlikely to outperform gray-scale because of the costs and feasibility to evaluate usually just one thyroid nodule per CEUS examination.

To conclude, CEUS is a promising noninvasive imaging method for the differential diagnosis of benign and malignant thyroid nodules. This study suggests that CEUS enhancement patterns were significantly different in benign and malignant lesions and that ring enhancement was very worthwhile in identification of benign lesions, whereas heterogeneous enhancement was valuable in detection of malignant lesions. The comparison of TIC shape in the nodule and in the surrounding tissue as a possible feature determining the biological nature of the lesion needs to be verified on a larger cohort of patients.

## Figures and Tables

**Table 1:** List of inclusion and exclusion criteria of the study.

Inclusion criteria
Presence of focal thyroid lesion greater than 8 mm
Willingness to undergo CEUS examination immediately after standard conventional US
Signed informed consent
Age over 18 years
Exclusion criteria
Cystic lesion of the thyroid gland with no solid component
Confusing terrain of multiple merging nodes occurring throughout the gland
Noncooperating patient
General contraindication for CEUS
Pregnancy

**Table 2:** Detailed description of qualitative parameters of CEUS.

Qualitative parameters of the CEUS enhancement pattern ^*∗*^	Description
Homogeneous	The entire lesion was homogeneously and diffusely enhanced at the time-of-peak intensity
Heterogeneous	The lesion was just partially or heterogeneously enhanced at the time-of-peak intensity
Ring enhancement	There was a rim-like enhancement around the lesion, which had a clear margin and could appear at the wash-in or wash-out phase. Once the peripheral rim appeared, the pattern would be the ring enhancement with no consideration of whether there were homogeneous or heterogeneous features
Absent	No enhancement: No microbubble echoes were detected within the lesion
**Time-intensity curves (TIC) shape of the nodule**	
Monophasic	Continuous gradual wash-out from the time of reaching peak intensity until the end of the second minute
Typical biphasic	Continuous gradual wash-out from the time of reaching peak intensity which is interrupted by a smaller second peak followed by further continuous wash-out until the end of the second minute
Atypical biphasic	The smaller peak during wash-in phase followed by a short period of wash-out and subsequent reaching of higher peak intensity followed by continuous gradual wash-out until the end of the second minute
Polyphasic	Polyphasic wash-out curve
**Comparison of the TIC shape of the lesion and the surrounding tissue**	
Identical	The same TIC shape in nodule and in surrounding tissue
Different	Different TIC shapes in nodule and in surrounding tissue

^*∗*^ Definitions of enhancement patterns in Appendix A are in concordance with the publication of Zhang et al. (2010) to keep uniformity of established methodology in our follow-up research.

**Table 3:** Detailed description of quantitative parameters of CEUS.

Quantitative parameters of CEUS	Description
Basic measurement	
Peak intensity (PI) (dB)	A parameter corresponding to the maximum achieved intensity
Time to peak (TTP)(sec)	Time from the moment of the contrast medium administration until the maximum intensity value is reached
Time from peak to one half (TFPTOH)(sec)	Time from the peak-intensity value to the half value of peak intensity during the wash-out period
Wash-in slope (WIS)(dB/sec)	A parameter represents how fast the contrast agent fills the selected region, indicating vascularization
**Derived indices**	
PI index	The ratio between the PI values from the ROI of the nodule and the ROI of surrounding thyroid tissue
TTP index	The ratio between the TTP values from the ROI of the nodule and the ROI of surrounding thyroid tissue
TFPTOH index	The ratio between the TFPTOH values from the ROI of the nodule and the ROI of surrounding thyroid tissue
WIS index	The ratio between the WIS values from the ROI of the nodule and the ROI of surrounding thyroid tissue
**Additional parameters**	
Echo mean—lesion peak	Echo mean in lesion at the time of peak
Echo mean—surrounding parenchyma	Echo mean in surrounding tissue at the time of peak
Echo standard deviation—lesion peak	Echo standard deviation in lesion at the time of peak
Echo standard deviation—surrounding parenchyma	Echo standard deviation in surrounding tissue at the time of peak
**Heterogeneity parameters**	
HVp	Heterogeneity value at the time of peak
HRp	Heterogeneity ratio at the time of peak

**Table 4:** Basic characteristics of analysed patients.

Characteristic sex	*N* = 68	%
Female	54	79.4
**Age**		
Median	54	
Range	26–80	
**Size of lesion** (mm)		
Median	24	
Range	9–99	
**Lesion composition**		
Solid	44	64.7
Cystic^*∗*^	0	
Spongiform	7	10.3
Mixed	17	25.0
**Biological nature**		
Benign	55	80.9
Malignant—papillary thyroid carcinoma	10	14.7
Malignant—follicular carcinoma	1	1.5
Malignant—DLBCL	1	1.5
Malignant metastasis—colorectal carcinoma	1	1.5
**Localization**		
Right lobe	35	51.5
Left lobe	29	42.6
Isthmus	4	5.9
**Reference method**		
Histopathology	48	70.6
FNA	20	29.4

^*∗*^ Purely cystic lesions were not included in this study.

**Table 5:** Results of CEUS qualitative parameters.

Qualitative characteristic count (percentage)	Benign *N* = 55	Malignant *N* = 13	*p* value
**Enhancement pattern**			<0.001
Homogeneous	24 (43.6%)	4 (30.8%)	
Heterogeneous	4 (7.3%)	9 (69.2%)	
Ring enhancement	27 (49.1%)	0	
Absent	0	0	
**TIC shape of the nodule**			0,275
Monophasic	21 (38.2%)	4 (30.8%)	
Typical biphasic	32 (58.2%)	7 (53.8%)	
Atypical biphasic	2 (3.6%)	1 (7.7%)	
Polyphasic	0 (0%)	0 (0%)	
Cannot be determined	0 (0%)	1 (7.7%)	
**Comparison of the TIC shape of the lesion and the surrounding tissue**			0.004
Identical	54 (98.2%)	9 (69.2%)	
Different	1 (1.8%)	3 (23.1%)	
Cannot be determined	0 (0%)	1 (7.7%)	

**Table 6:** The ROC analysis of quantitative characteristics.

ROC analysis of quantitative characteristics	AUC (%)	*p* value	Cut-off ^*∗*^	Sensitivity (%)	Specificity (%)	Overall accuracy (%)
PI	50.8	0.467	6.5	58.3	64.8	63.6
TFPTOH	60.6	0.876	34.5	83.8	55.6	60.6
WIS	54.5	0.688	1.4	66.7	50.0	53.0
TTP	57.1	0.780	13.8	100	22.2	36.4
PI index	50.1	0.506	1.32	30.8	81.8	72.1
TFPTOH index	63.9	0.941	1.21	38.5	89.1	79.4
WIS index	51.6	0.432	0.56	30.8	89.1	77.9
TTP index	50.1	0.500	1.03	84.6	30.9	41.2
HVp	59.7	0.854	147.2	41.7	79.6	72.7
HRp	50.8	0.467	0.88	33.3	81.5	72.7

^*∗*^ TFPTOH, WIS, TTP values, PI index, TFPTOH index, and HVp values greater than or equal to the cut-off were considered malignant. In contrast, for PI values, WIS index, TTP index, and HRp parameter, values less than or equal to the cut-off were considered malignant.

## Data Availability

The data are available on request to the first author Hana Petrasova (petrasova.hana@fnbrno.cz).

## Abbreviations

AUC: Area under the curve
CEUS: Contrast-enhanced ultrasound
DLBCL: Diffuse large B-cell lymphoma
EFSUMB: The European Federation of Societies for Ultrasound in Medicine and Biology
FNA: Fine-needle aspiration
HR: Heterogeneity ratio
HRp: Heterogeneity ratio at the time of peak
HV: Heterogeneity value
HVp: Heterogeneity value at the time of peak
NPV: Negative predictive value
PI: Peak intensity
PPV: Positive predictive value
ROC: Receiver operating characteristic
ROI: Region of interest
TFPTOH: Time from peak to one half
TIC: Time-intensity curves
TTP: Time to peak
WIS: Wash-in slope.

## Appendix

### A. Detailed Description of Qualitative Parameters of CEUS

A detailed description of qualitative parameters of CEUS is listed in Table 2.

### B. Software Functions Used for Quantitative Evaluation of CEUS

To obtain the most objective values of CEUS quantitative evaluation, the following software functions were used (Tables 2, 3, and 6).

Active motion compensation©—the motion compensation algorithm attempts to overcome artifacts arising from the periodic motion of the examined organ, such as respiration and pulsation of surrounding large vessels, to provide the best quality data for quantification.

Set background©—this function of subtraction of nonzero background signal intensity of the examined area before contrast enhancement itself eliminates the influence of default nonzero setting of image gain parameter before contrast application and thus removes the falsely higher absolute value of postcontrast intensity, and only true signal increment after contrast application is evaluated. From the recorded video loop, the frame just before the contrast media application was selected as the background frame, which results in the subtraction of the corresponding value of the data point from every data point available in the waveform.

### C. Detailed Description of Quantitative Parameters of CEUS

A detailed description of quantitative parameters of CEUS is listed in Table 3.

### D. Table Demonstrating the ROC Analysis of Quantitative Characteristics

Table 6 demonstrates the ROC analysis of quantitative characteristics.
